# Complete mitochondrial genome of *Morphostenophanes sinicus* (Zhou, 2020) (Insecta: Coleoptera: Tenebrionidae)

**DOI:** 10.1080/23802359.2021.1970636

**Published:** 2021-09-15

**Authors:** Yu Bai, Xuyuan Gao, Yonghao Yu, Xiuzhen Long, Xianru Zeng, Dewei Wei, Lin Ye

**Affiliations:** a College of Mathematics & Information Science, Guiyang University, Guiyang, China; b Guangxi Key Laboratory of Biology for Crop Diseases and Insect Pests, Institute of Plant Protection, Guangxi Academy of Agricultural Sciences, Nanning, China; c College of Biology and Environmental Engineering, Guiyang University, Guiyang City, China

**Keywords:** *Morphostenophanes sinicus*, Tenebrionidae, mitochondrial genome, phylogenetic analysis, mitogenome

## Abstract

The genus *Morphostenophanes* is a small arboreal group of darkling beetles that are endemic to the Oriental region. The complete mitochondrial genome of the *M. sinicus* population from the Manwan Town was first characterized. The mitogenome consisted of a circular DNA molecule of 15,662 bp with a 66.352% AT content. It comprises 13 protein-coding genes (PCGs), 22 tRNA genes, and two rRNA genes. The PCGs have a typical ATN (Met) start codon, except for *nad1* (TTG as a start codon), and are terminated by typical TAN stop codons.

The genus *Morphostenophanes*, which is a small arboreal group of darkling beetles endemic to the Oriental region (Gao and Ren 2009), was established when *Morphostenophanes aenescens* (Pic, 1925) was collected from Yunnan, China (Zhou 2020). Similar to *M. gaoligongensis*, *M. sinicus* (Zhou 2020) is distributed in Yunnan, is bronze in color with a green sheen, shagreened, dark brown antennae, and reddish-brown claws (Zhou 2020). Therefore, the complete mitogenome of the *M. sinicus* population from the Manwan Town was characterized, to elucidate the molecular evolution and taxonomic affinities of the genus *Morphostenophanes*.

Adult *M. sinicus* specimens were collected from Manwan Town, Yunnan Province, China, on 24 September 2020 (geographic location: 100.32963 N, 24.68407 E). The specimen was deposited by the corresponding author in the insect specimen room of the Institute of Plant Protection, Guangxi Academy of Agricultural Sciences and given a specimen accession number (GIPP-20200924-002). Genomic DNA was isolated and fragmented to generate a genomic library, using the VAHTS^®^ Universal DNA Library Prep Kit for MGI (Vazyme, Nanjing, China). Sequencing was subsequently performed on the MGI-SEQ 2000 platform (MGI Tech Co., Ltd., Shenzhen, China), using an insert size of 400 bp. We obtained approximately 9.179 Gb raw data (2 × 150 bp paired-end reads), of which 9.046 Gb (98.548%) was high-quality, clean data. The genome was assembled *de novo* using SOAPdenovo v2.04 (https://github.com/aquaskyline/SOAPdenovo2/) (Luo et al. 2012) and MITObim v1.6 (https://github.com/chrishah/MITObim) (Hahn et al. 2013).

The mitogenome of *M. sinicus* (GenBank accession number: MW853764) consists of a circular DNA molecule that is 15,662 bp (genome nucleotide composition: 37.856% A, 28.496% T, 21.995% C, and 11.652% G; 66.352% AT content). Using Perna and Kocher’s formula (Perna and Kocher 1995), the AT- and GC-skews of the major strand of the mitogenome were calculated to be 0.141 and −0.307, respectively. The AT-rich region of the mitogenome was 1105 bp, with a 78.733% AT content, and is located between the genes encoding srRNA and tRNA-Ile.

The mitogenome of *M. sinicus* contains 13 protein-coding genes (PCGs), 22 tRNA genes, and two rRNA genes, which were annotated using the MITOS Web Server (http://mitos.bioinf.uni-leipzig.de/) (Bernt et al. 2013). The transcriptional start and stop sites of the protein coding regions were manually corrected using *Tenebrio obscurus* (Bai et al. 2018), *Zophobas atratus* (Bai et al. 2019), *Blaps rhynchoptera* (Yang et al. 2019), and *Promethis valgipes valgipes* (Bai et al. 2021) as references. All 13 PCGs had typical ATN (Met) start codons, except for *nad1* that had a TTG start codon. Four genes (*nad2*, *nad3*, *nad5*, and *nad6*) had an ATA start codon, one (*atp8*) had an ATC start codon, six (*cox1*, *atp6*, *cox3*, *nad4*, *nad4l*, and *cob*) had an ATG start codon, and one (*cox2*) had an ATT start codon. All 13 PCGs had typical TAN stop codons: six genes (*nad2*, *atp8*, *atp6*, *nad4l*, *nad6*, and *cob*) had TAA stop codons, three (*cox2*, *nad3*, and *nad1*) had TAG stop codons, and four (*cox1*, *cox3*, *nad5*, and *nad4*) had an incomplete stop codon, consisting of a codon that was completed by the addition of A nucleotides at the 3′ end of the encoded mRNA. The 22 tRNA encoding genes were interspersed throughout the coding region and ranged from 60 bp (tRNA-Cys and tRNA-Tyr) to 70 bp (tRNA-Lys). The genes encoding lrRNA and srRNA were 1214 and 750 bp, respectively. A maximum overlap length of 7 bp was located between *nad4* and *nad4l*, *atp8*, and *atp6*. The maximum length of the intergenic region was 32 bp, located between lrRNA and tRNA-Val.

To validate the phylogenetic position of *M. sinicus*, its mitochondrial PCGs, and those of 14 other species of Insecta, were used to construct a maximum-likelihood phylogenetic tree using the MEGA X software (Kumar et al. 2018) (Figure 1). Thirteen mitogenomes were downloaded from NCBI, which belong to Tenebrionidae, and the *Lepisma saccharina* mitogenome (Bai et al. 2020) was selected as the outgroup. Based on Bayesian information criterions (BICs), the General Reversible Mitochondrial (mtREV24) model with amino acid frequencies (+F), gamma distribution (+G, parameter = 0.5107) with five rate categories, and evolutionarily invariable sites (+I, 23.53%) was chosen as the optimal evolutionary model, with 1000 bootstrap replicates, for phylogenetic analysis. *M. sinicus* is closely related to *Asbolus verrucosus* (Rider 2016) and *Adelium* sp. NCS-2009 (Sheffield et al. 2009). Overall, our study provides insights into the mitogenome of *M. sinicus*, providing essential genetic and molecular data for further phylogenetic and evolutionary analyses of the Tenebrionidae.

**Figure 1.**
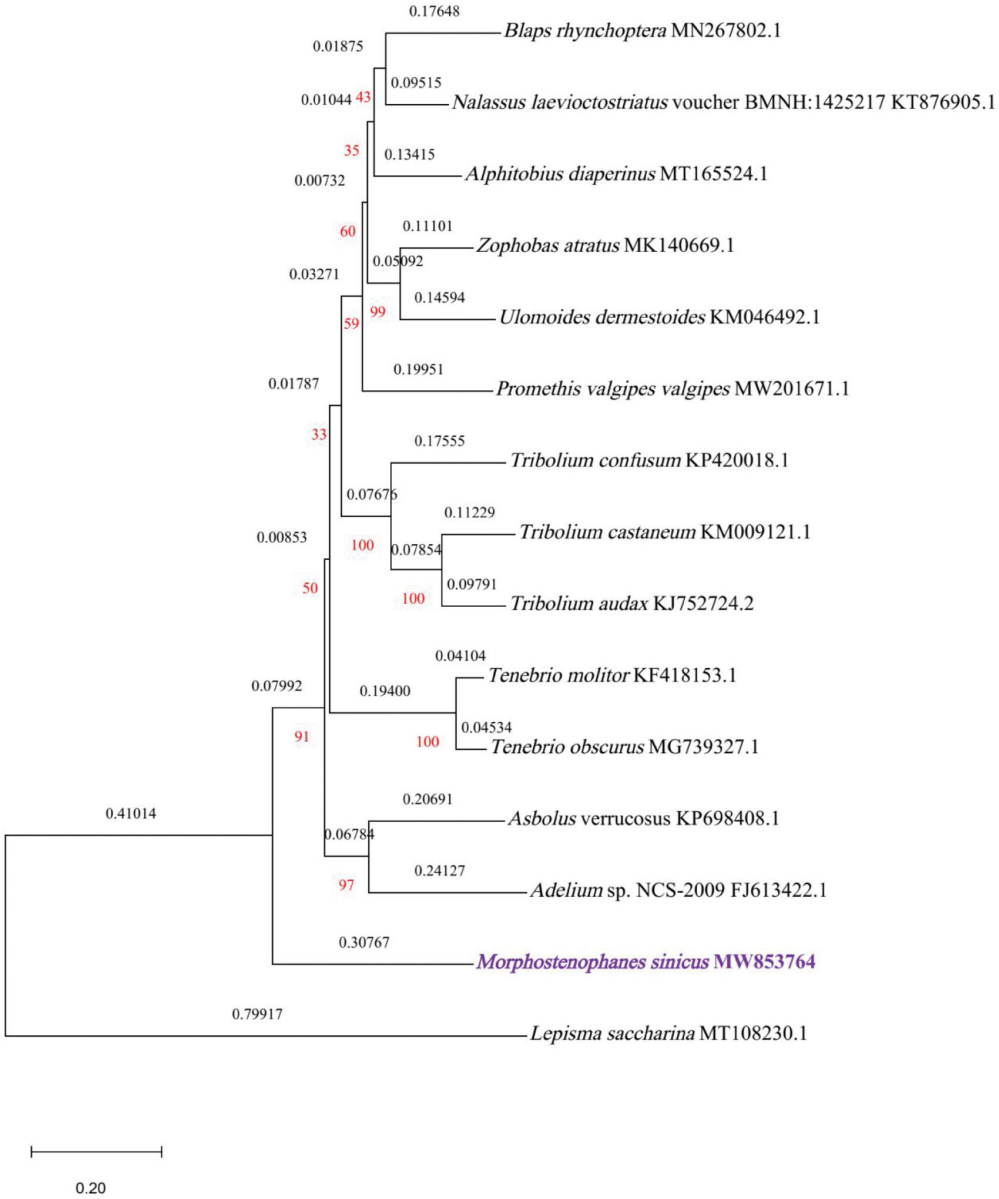
Maximum-likelihood phylogenetic tree of *Morphostenophanes sinicus* and 14 other species of Insecta based on 13 protein-coding regions of their mitogenomes.

Phylogenetic relationships were inferred using maximum likelihood, and the general reversible mitochondrial + Freq. model. The tree with the highest log likelihood (–44628.24) is shown. The percentage of trees in which the associated taxa were clustered together is shown below the branches. The initial tree(s) for the heuristic search were obtained automatically by applying Neighbour-Join, and BioNJ algorithms to a matrix of pairwise distances estimated using a JTT model, and then selecting the topology with a superior log likelihood value. A discrete gamma distribution was used to model evolutionary rate differences among sites (five categories (+G, parameter = 0.4640)). The rate variation model allowed for some sites to be evolutionarily invariable ((+I), 23.53% sites). The tree is drawn to scale, with branch lengths representing the number of substitutions per site (above the branches). The percentage of trees in which the associated taxa were clustered together is shown in red. This analysis involved 15 amino acid sequences. The complete mitogenome of *M. sinicus* determined in this study is indicated in purple.

## Data Availability

Mitogenome data supporting this study are openly available in GenBank at https://www.ncbi.nlm.nih.gov/nuccore/MW853764. SRA records were accessible with the following link https://www.ncbi.nlm.nih.gov/sra/PRJNA719484.
